# Three Draft Genome Sequences of *Vibrio coralliilyticus* Strains Isolated from Bivalve Hatcheries

**DOI:** 10.1128/genomeA.01162-17

**Published:** 2017-10-12

**Authors:** Hanna Kehlet-Delgado, Gary P. Richards, Claudia Häse, Ryan S. Mueller

**Affiliations:** a Department of Microbiology, Oregon State University, Corvallis, Oregon, USA; b U.S. Department of Agriculture, Agricultural Research Service, Dover, Delaware, USA; c Department of Biomedical Sciences, College of Veterinary Medicine, Oregon State University, Corvallis, Oregon, USA

## Abstract

Reported here are the genome sequences of three *Vibrio coralliilyticus* isolates RE87, AIC-7, and 080116A. Each strain was isolated in association with oyster larvae in commercial aquaculture systems. These draft genomes will be useful for further studies in understanding the genomic features contributing to *V. coralliilyticus* pathogenicity.

## GENOME ANNOUNCEMENT

*Vibrio coralliilyticus* is a known pathogen of corals (1) and shellfish, including Pacific oyster (*Crassostrea gigas*) larvae (2–5). In order to further the understanding of the genomic repertoire of *V. coralliilyticus*, particularly isolates pathogenic to oyster larvae, we sequenced three strains isolated from oyster aquaculture facilities. Strain RE87 was isolated in 2000 from dying *C. gigas* seed at a Hawaii nursery. Strain AIC-7 was isolated from a vibriosis outbreak among Eastern oysters (*Crassostrea virginica*) in 2015 at a hatchery in New Jersey. Strain 080116A was isolated in 2016 from *C. gigas* larvae tanks at an Oregon hatchery. All three strains demonstrated a degree in pathogenicity in preliminary pathogenicity challenges with *C. gigas* larvae (80 to 100% larval mortalities after 48 h [our unpublished data]).

DNA from overnight cultures was isolated and the Nextera XT kit (Illumina) was used to prepare libraries for sequencing on a MiSeq Illumina platform at the Center for Genome Research and Biocomputing at Oregon State University. Each genome library was sequenced with 250-bp paired-end reads at an estimated minimum coverage of 65-fold. The resulting reads were trimmed and quality filtered (*Q* > 28, minimum read length 150 bp) using Sickle (6) and randomly subsampled to an estimated coverage of 50-fold. Reads were subsequently *de novo* assembled using IDBA-UD (7). The assembly for strain RE87 had a genome size of 5.59 Mbp (45.8% GC), 49 contigs (*N*_50_ = 335,391 bp), and a maximum contig size of 1,074,445 bp; the assembly for strain AIC-7 resulted in a genome size of 5.95 Mbp (45.3% GC), 65 contigs (*N*_50_ = 274,060 bp), and a maximum contig size of 1,201,114 bp; and the assembly for strain 080116A had a genome size of 5.63 Mb (45.7% GC), 107 contigs (*N*_50_ = 275,484 bp), and a maximum contig size of 1,249,611 bp.

Gene predictions for the draft assemblies were performed using Prokka version 1.11 (8) and Rapid Annotation using Subsystem Technology (RAST) (9). There were 5,145, 5,542, and 5,205 coding sequences detected for strains RE87, AIC-7, and 080116A, respectively. Proteinortho (10) detected 1,813 orthologous clusters that were shared among the three isolates, representing 57 to 62% of the predicted proteome of each genome. Strain RE87 has 266 unique genes, AIC-7 has 504, and 080116A has 287. To compare genome similarities with other *V. coralliilyticus* strains, average nucleotide identities (ANI) were calculated using Enviomics (11). ANI values for each strain with *V. coralliilyticus* strains ATCC BAA-450 (12), RE98 (13), OCN014 (14), and 58 (15) were >96%, corresponding to grouping at the species level (16, 17). All three strains had open reading frames displaying 99% homology to the *VtpA* extracellular zinc metalloprotease of *V. coralliilyticus* RE98, a known virulence factor for *C. gigas* (18). A more in-depth genomic analysis of these and other *V. coralliilyticus* strains will be conducted for a future publication.

### Accession number(s).

This whole-genome shotgun project has been deposited in GenBank under the accession numbers NRHY00000000 (RE87), NRQO00000000 (AIC-7), and NRHV00000000 (080116A). The versions described in this paper are the first versions.
